# Pilot evaluation of an educational and lived experience training program on ketogenic metabolic therapy in mental health care for health care professionals

**DOI:** 10.3389/fpsyt.2026.1913325

**Published:** 2026-09-22

**Authors:** Sidney L. Murray, Denise Potter, Yaara Zisman-Ilani, Hwiyoung Lee, Shuo Chen, Daniel J. O. Roche, Laura M. Rowland, Christopher M. Palmer, Deanna L. Kelly

**Affiliations:** 1 Maryland Psychiatric Research Center, University of Maryland School of Medicine, Baltimore, MD, United States; 2 Advanced Ketogenic Therapies, Limited Liability Company (LLC), Petersburg, MI, United States; 3 Department of Social & Behavioral Sciences, Barnett College of Public, Temple University, Philadelphia, PA, United States; 4 Department of Psychiatry & Behavioral Science, Lewis Katz School of Medicine, Temple University, Philadelphia, PA, United States; 5 Department of Clinical, Educational & Health Psychology, Division of Psychology & Language Sciences, University College London, London, United Kingdom; 6 Metabolic and Mental Health Program, McLean Hospital and Harvard Medical School, Belmont, MA, United States

**Keywords:** competence, shared decision-making, health care professionals, ketogenic diet, mental health, training model, ketogenic metabolic therapy, education

## Abstract

**Background:**

Serious mental illness imposes a substantial burden. Pharmacologic treatments remain only partially effective and are associated with significant side effects, highlighting the need for novel treatment approaches. Ketogenic metabolic therapy (KMT) is emerging as an intervention for serious mental illness, with a growing evidence base and strong patient interest. A critical barrier to KMT implementation is the lack of health care professional (HCP) competence and readiness to discuss KMT with patients with serious mental illness. Existing training does not adequately address HCP competence in presenting KMT as a treatment option. This study describes the pilot-testing of “Live It, Launch It” (LI-LI), the first KMT training program designed to build self-reported competence to discuss KMT in psychiatric care for HCPs.

**Methods:**

LI-LI combines a 4-hour asynchronous educational course on KMT with a 4-week experiential group KMT intervention during which participants adhered to a ketogenic diet. LI-LI aimed to improve self-reported competence to utilize KMT and perceived ability to engage in shared decision-making with patients about incorporating KMT into their treatment plan.

**Results:**

Forty HCPs participated in the education course: 10 psychiatrists, 10 psychiatric pharmacists, 8 psychologists/therapists, and 12 other HCPs (e.g., nurse practitioners, physician assistants). Twenty-five initiated the diet intervention. Baseline competence scores (visual analog scale 0-100) were low, M(SE) = 17.40 (2.68), increasing to M(SE) = 68.08 (2.47) after education (d = 2.88, p <0.0001), and to M(SE) = 86.62 (1.82) post-diet (d = 1.08), with a very large whole-study effect (d = 3.49, p < 0.0001). Shared decision-making scores at baseline were M(SE) = 53.64 (2.89), increasing to M(SE) = 83.35 (1.83) after the educational course (d = 1.74, p <0.0001), and to M(SE) = 91.23 (2.05) after the diet phase (d = 0.73, p = 0.009), with a very large whole-study effect (d = 2.53, p < 0.0001). Feedback indicated that lived experience with KMT was helpful for implementation-readiness.

**Discussion:**

The educational course on KMT was associated with increased self-reported competence and perceived shared decision-making in HCPs, and this was enhanced with combined education and lived experience. Implementation of this approach could accelerate KMT into clinical care. Results should be interpreted with caution as this was a small uncontrolled study with self-reported main outcomes.

**Clinical trial registration:**

## Background

According to the World Health Organization (WHO), nearly 1 billion people worldwide were living with a mental disorder in 2019, underscoring the vast global burden of mental illness (1). The economic impact is similarly profound; for example, the societal cost of schizophrenia alone in the United States was estimated at $366.8 billion in 2024 (2). Existing pharmacologic treatments are often only partially effective and frequently associated with significant adverse effects (3–12). These adverse effects contribute to poor adherence or treatment discontinuation, increasing the risk of relapses and suicide (11, 13, 14). In addition, current clinical guidelines provide limited support for individualized medication selection partially due to lack of understanding underlying pathophysiology and disease subgroups (15). As a result, psychopharmacologic treatment often relies on a trial-and-error approach to balance therapeutic benefits and tolerability (16).

Individuals with serious mental illness experience disproportionately high rates of medical comorbidities, including obesity, type 2 diabetes, cardiovascular disease, and metabolic syndrome, all of which contribute substantially to disability, reduced quality of life, and escalating healthcare expenditures (17–19). These comorbidities arise from such factors as social determinants of health, lifestyle choices, chronic stress, psychotropic medication effects, and potentially shared biological mechanisms linking metabolic and psychiatric dysfunction (20, 21). As a result, people with serious mental illness die 10–20 years earlier than the general population, with the majority of mortality attributable not to suicide, but to preventable and treatable chronic medical illnesses, particularly cardiometabolic disease (22). In addition to the significant human burden, these overlapping psychiatric and medical conditions contribute to markedly increased healthcare utilization and economic costs across inpatient, outpatient, emergency, and long-term care settings (19, 23).

The limited efficacy of available treatments, along with the need to simultaneously address physical health comorbidities, have driven efforts to identify novel and more acceptable treatment options. Increasingly, systems, patients, families, and caregivers are requesting focus and attention on alternative and holistic treatments beyond or in addition to traditional pharmacologic interventions (24–30).

One emerging potential treatment is the use of a ketogenic diet for medical treatment, also referred to as ketogenic metabolic therapy (KMT). This growing body of evidence suggests that metabolic dysfunction may contribute to psychiatric disorders and that the state of ketosis may represent a novel therapeutic mechanism for treatment (31, 32). KMT is a well-established treatment for epilepsy, with over a century of supporting evidence (33, 34). In the context of KMT for epilepsy, macronutrient ratios are typically 3:1 or 4:1 grams of fat to combined protein and carbohydrates (34). Emerging data on KMT in psychiatric populations, is being used in modified ratios typically between 1:1-2.5:1 grams of fat to combined protein and carbohydrates (see 35, 36-40).

Early studies using KMT for mental illness show promising symptom improvements in schizophrenia, bipolar disorder, and depression (35–42). The first randomized controlled trial for KMT in serious mental illness has recently been published, which found metabolic improvements in participants with bipolar and schizophrenia after 1 month on KMT compared to diet-as-usual controls, and found significant improvement in positive, negative, cognitive, and depressive symptoms in participants on KMT for 4-months (36). Importantly, the study phase that showed significant psychiatric improvement was single-arm (36). Therefore, we still need randomized controlled trials testing psychiatric improvement on KMT for sufficient durations of time in order to have gold standard evidence for KMT in psychiatric care. Another randomized controlled trial that is designed to do this is currently finalizing (43). In addition to psychiatric improvement, KMT may be useful in attenuating common metabolic health comorbidities observed in people with serious mental illness, such as metabolic syndrome, insulin resistance, and type 2 diabetes (44–46).

While further research is needed, clinical recommendations have recently been published supporting the use of KMT alongside standard care in psychiatry (47). However, preparation for improving knowledge about KMT will likely be insufficient to change prescribing and implementation behaviors. Many treatments in psychiatry, despite an adequate evidence-base, remain slow to be adopted and often remain underutilized, as is the case with clozapine, long-acting injectable antipsychotics and lithium, three effective and underused treatments (48–51). Barriers for implementation into practice are multifactorial but often include the fact that health care professionals (HCPs) report lack of competence/confidence in using new or more complex treatments (52–54). Changing prescribers’ behaviors has been a major challenge and scientific focus using many traditional educational approaches, such as dissemination of printed educational materials, academic detailing, and continuing education workshops to improve knowledge of the topic. Nonetheless, these strategies have been shown to be insufficient in changing clinician behavior or improving patient outcomes (55, 56). More active strategies such as audits and feedback, or educational outreach visits, show only modest effects in changing behaviors beyond educational strategies and remain limited in their impact (57, 58).

Successful implementation of a new treatment modality into practice depends on three key elements: evidence, context, and facilitation (59). Facilitation involves the processes and strategies used to guide and support HCPs throughout the implementation process, helping to overcome barriers and promote the successful adoption of the new treatment. These elements are strengthened through immersive, or hands-on experiential learning approaches which have been shown to improve learner engagement, knowledge, retention, and confidence, the latter which is known to be a more reliable predictor of behavior change (60–62). Educational theory further emphasizes the importance of self-efficacy in promoting behavior change. Self-efficacy refers to an individual’s belief in their ability to successfully perform a task such as prescribing or using new treatments (63). Closely related to this concept is competence, or a clinician’s perceived ability to implement a task, which serves as an important mediator or driver of behavior change and adoption of new practices (64, 65). Our previous work has focused on strategies to improve competence for improving psychiatric treatment implementation (66).

We therefore investigated whether an educational program incorporating active participation and lived experience designed to facilitate how to plan, initiate, and monitor a ketogenic diet, would improve HCP self-reported competence for using a ketogenic diet with patients. “Live it Launch it” (LI-LI) is a training model designed to enhance HCP self-reported competence and confidence in shared decision making with their patients specifically with regards to implementing KMT for mental illness. Given growing evidence, patient demand and limited HCP training, this study aimed to assess whether immersive education could improve HCP self-reported competence (rather than objective assessment) to incorporate this emerging treatment into clinical practice.

## Methods

### Design

This study was a single-arm open-label pilot trial that employed a two-part training model. The first component consisted of an online educational intervention about KMT for mental health (described in **Supplementary Table 1**) (educational phase), and the second phase was a 4-week lived cohort experience in which participants implemented the ketogenic diet themselves (diet phase). This yielded a total of 3 study timepoints (pre-education/baseline, post-education/midpoint, and post-diet/endpoint).

### Participants

HCPs from across the United States were recruited, and the first 40 eligible individuals were enrolled. Eligibility criteria for the educational component included age 18–74 years, not currently following a ketogenic diet, no prior successful personal implementation of a medical ketogenic diet, and active involvement in clinical care or research as an HCP. Target enrollment was N = 30 but permitted up to N = 40. Forty participants completed the educational phase, and 21 of these participants also completed the diet phase.

Eligibility for participation in the dietary intervention included ages 18–74 years, body mass index >18.5 (self-reported), not currently on KMT, and no prior successful experience implementing a ketogenic diet. Exclusion criteria included pregnancy or lactation, type 1 diabetes, insulin-dependent type 2 diabetes, or diagnosed carnitine deficiency.

All participants met with a research coordinator either in person or via Zoom to review study procedures and provide informed consent. Participants completed the Evaluation to Sign Consent (ESC) to assess decisional capacity, and written informed consent was obtained electronically (Docusign^®^) or in person. The study protocol was approved by the University of Maryland Institutional Review Board.

## Materials

### Educational coursework

All participants were asked to complete the same educational program. The educational content was delivered through the Ketogenic Metabolic Therapy for Psychiatric Conditions course (67). Specifically, this was a 4-hour asynchronous course consisting of four modules covering foundations of KMT such as its history and illness application and how glucose, ketones, and insulin work; variations and personalization of KMT such as the classic versus modified ketogenic diet; how to do a well-formulated ketogenic diet, which aims to optimize overall nutrition on the ketogenic diet; and ketogenic management, such as monitoring labs and addressing adverse effects (See **Supplementary Table 1**). The course also included a substantial library of optional supplemental resources, including quizzes, scientific literature, case studies, patient interviews, educational videos, implementation guides, food lists, KetoDietCalculator tutorials, resource directories, and additional condition-specific learning materials. This course is available to the public for $299 but was provided at no cost to participants as part of the study. No continuing medical education (CME) credits were offered; however, continuing education units (CEU) were available for Registered Dietitian Nutritionists (RDNs).

### Diet intervention

After a 2-hour meeting with an RDN or nutrition specialist physician, participants were instructed to initiate a medical ketogenic diet with a target macronutrient ratio ~1.5:1 (grams of fat to combined grams of carbohydrate and protein), but could range from 1:1 to 2.1, depending on individual ability to reach ketosis. Individual daily caloric requirements were calculated in collaboration with an RDN or nutrition specialist physician, incorporating age, sex, weight, height, activity level, and individual goals (e.g., maintaining vs. losing weight). Macronutrient ratios and caloric targets were adjusted throughout the intervention to optimize ketosis, support weight goals, and ensure adequate protein intake. Participants were able to choose between a precision ketogenic diet, in which a gram scale is used to weigh food, or a modified ketogenic diet, in which measuring cups and measuring spoons are used.

Participants were responsible for implementing the diet independently using standardized guidance provided during the educational program, supplemented by individualized recommendations from the RDN or nutrition specialist physician. All participants began the intervention within the same 7-day window and followed dietitian-prescribed caloric targets (e.g., 2000–2200 kcal/day). Nutritional plans were individualized and ranged from two meals per day to three meals and 2 snacks per day. Ongoing support was provided by the lead RDN, who assisted with calculating macronutrient distributions and making iterative adjustments during the study period.

Participants tracked dietary intake using the Cronometer^®^ application (Gold version), which was provided to them at no cost (68). The goal was to achieve and maintain nutritional ketosis, defined as blood β-hydroxybutyrate (βHB) levels ≥0.5 mmol/L, with an expected range of 1–5 mmol/L for most participants. To track βHB, KetoMojo^®^ glucose and ketone monitors were provided to participants at no cost. Food scales were provided as needed to support accurate measurement of intake, also at no cost to participants. More details on diet-related materials will be provided in another publication.

Participants were encouraged to review medications for carbohydrate content, particularly liquid formulations. Reporting medications was optional to respect participant privacy. A daily multivitamin was recommended, and additional supplementation (e.g., vitamin D, calcium) was advised at the discretion of the RDN or nutrition specialist physician. Further dietary details and specific health outcomes using the diet, such as macronutrients, micronutrients, βHB, health outcomes, and side effects, will be reported in another paper.

### Clinical assessments

At baseline, participants completed demographic information and two self-efficacy-based assessments.

### Self-reported competence

The primary outcome was self-reported competence. This construct is grounded in self-efficacy and defined in this study as a participant’s perceived ability to implement KMT in patients with mental illness (65). Consistent with established measurement approaches for assessing self-reported competence as described above and our experience using this as an outcome in educational interventions (65, 66, 69), we assessed self-reported competence using a Visual Analog Scale (VAS) ranging from 0–100 mm, with anchors of 0 (no competence) and 100 (fully competent). Overall self-reported competence was measured by the question “How competent are you at implementing a ketogenic diet?” Additionally, 14 additional sub-items were asked relating to various specific aspects of the process, including monitoring ketones, assessing side effects, etc. (Full questions are in **Supplementary Table 2**). Self-efficacy ratings have demonstrated strong reliability and validity in assessing perceived competence (61, 70, 71).

### Self-efficacy of shared decision making

A secondary outcome was perceived shared decision making ability with patients, which refers to HCPs’ level of confidence in supporting their patients in making an informed choice and using a ketogenic diet in the future. The Decision Self-Efficacy Scale (DSE), a validated and reliable 11-item self-report instrument (72–74), was used to evaluate confidence in shared decision making. The DSE is rated on a 5-point Likert-type scale ranging from 0 (not at all) to 4 (very confident). This measure was used to evaluate HCP readiness to incorporate KMT into patient-centered care discussions. See the list of items in **Supplementary Table 3**.

## Exploratory qualitative data

At the conclusion of the study, we asked if participants thought that LI-LI improved their ability to implement a ketogenic diet. We also asked questions on what aspects of learning helped them the most, what aspects surprised them, and what they would change about the educational program. Additionally, many participants also included many discussion points they would want to emphasize with their patients. We also asked about clinical populations for which participants were interested in using KMT.

All study data were collected and managed using the REDCap system. Participants were asked open-ended questions regarding the most helpful aspects of the program to collect rich qualitative data that may pick up information the questionnaires did not. Qualitative analysis was conducted in accordance with methods for thematic analysis by Braun & Clarke (2006) and following the specific steps outlined by Ahmed (2025). Broadly, responses were read multiple times and initial impressions were noted; initial codes were generated upon later reads; candidate themes were recorded; and final themes were chosen (See **Supplementary Table 4**).

### Procedure

After providing informed consent, participants received a link to complete baseline assessments, including measures of self-reported competence and shared decision making. These questionnaires were administered through REDCap. After participants completed baseline assessments, the educational intervention began. The education phase included 4-hours of online asynchronous course curriculum; two 1.5-hour educational sessions available to all national course participants; and three weekly one-hour free form Q&A sessions with the lead Registered Dietitian Nutritionist (RDN), which were provided exclusively to LI-LI study participants following diet initiation. Participants were given approximately one month to complete baseline assessments, online training, and post-education assessments of self-reported competence and shared decision making for future use with patients. After completing the post-education assessments, participants were invited to continue into the experiential diet phase of the study and implement KMT themselves as part of a cohort experience (living out the diet). This phase consisted of an initial 1–2-hour individual consultation with a dietitian or nutrition specialist physician, followed by a 4-week self-implemented ketogenic diet as a cohort. During this period, participants were invited to attend a weekly live group-based Q&A with the lead study dietitian and each other to ask questions about the diet, share recipes and strategies, and ask about side effect management. After the 4-week diet experience, participants completed endpoint assessments of self-reported competence and shared decision making. More details on the diet phase of the study, including food tracking, health outcomes, detailed side effect coverage, and qualitative feedback on living out the diet, will be published elsewhere.

### Analysis of data

This pilot trial was designed to inform a larger implementation study. The pre-to-post educational study included the sample of N = 40, and pre-to-post-diet changes included the diet completers (N = 21). Pre-to-post changes in 100 mm VAS scores measuring self-reported competence for the educational program as well as perceived shared decision making using the DSE scale were calculated using linear mixed-effects models. Time was included as a categorical fixed effect with three levels, and a participant-specific random intercept was included to account for within-participant correlation among repeated measurements. Under the assumptions of independent and homoscedastic residual errors, this mixed-effects model specification induces a compound-symmetry marginal covariance structure within participants. Models were estimated using restricted maximum likelihood (REML), with degrees of freedom approximated using the Satterthwaite method. All available observations were included without restricting the analyses to participants with complete data at all time points. Follow-up pairwise comparison between time points were conducted using estimated marginal means. Qualitative responses were analyzed using thematic analysis (75), and the 16-item checklist for this process was followed to ensure rigorous application of this method (76).

### Results

The study opportunity was posted using an IRB approved graphic via the Maryland Psychiatric Research Center (MPRC) social media pages (LinkedIn, Instagram and X) and reached full enrollment (N = 40) within two weeks. One participant withdrew shortly after consenting due to an unrelated hospitalization and was replaced prior to study initiation. A CONSORT diagram is presented in **Figure 1**. Of the 40 enrolled participants, 26 elected to continue into the diet phase. Reported reasons for non-participation in the diet phase included pregnancy or plans for pregnancy (n=4), scheduling conflicts (n=4), unspecified reasons (n=4), dietary restrictions (n=1), and concerns about side effects (n=1). One was excluded from the diet phase before it began due to low BMI.

**Figure 1:**
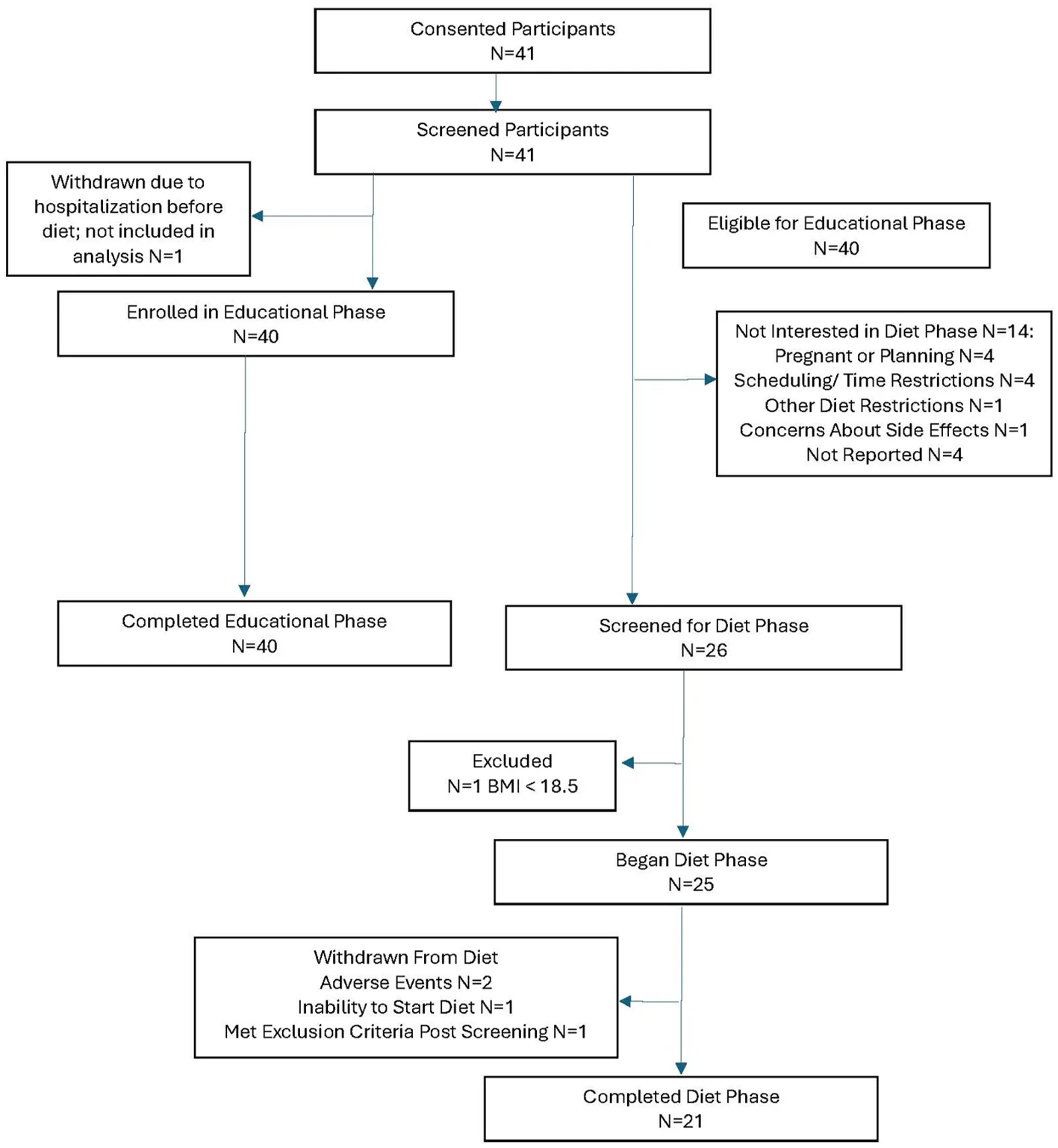
Participant flow CONSORT diagram.

The sample included approximately 25% physicians, 25% pharmacists, 20% psychologists/therapists, and 30% other health professionals (including nurses, physician assistants, nurse practitioners, and researchers). All participants (N = 40) completed the educational component. See **Table 1** for participant demographics. Among those entering the diet phase, 21 of 25 participants (84%) completed the full 4-week intervention. One participant was excluded after enrollment for not meeting eligibility criteria; one was unable to start with the cohort; and two were withdrawn due to adverse events, which were believed to be related to the diet and were rated as moderate (muscle soreness, heart palpitations). The person with muscle soreness withdrew after 3 weeks on the diet and did not reach resolution by the end of the 4-week study despite diet discontinuation the week prior. The person with heart palpitations withdrew after 2 weeks on the diet and reported resolution after discontinuing the diet and medium-chain triglycerides (MCT) oil. Diet benefits, tolerability, adverse effects, and laboratory values associated with the diet in N = 21 will be discussed in further detail in another publication.

**Table 1:** Demographic information.

	Education phase (N = 40)	Diet phase (N = 21)
*Sex*	*N (%)*	*N (%)*
Female	29 (72.5%)	15 (71%)
Male	11 (27.5%)	6 (29%)
Race		
Black	5 (12.5%)	2 (10%)
White	30 (75%)	16 (76%)
Asian	4 (10%)	2 (10%)
Multiracial	1 (2.5%)	1 (5%)
Profession		
Pharmacists	10 (25%)	7 (33%)
Physicians	10 (25%)	2 (10%)
Psychologists/Therapists	8 (20%)	3 (14%)
Other	12 (30%)	9 (43%)
Age	Mean (SD)	Mean (SD)
	44.41 (9.16)	46.59 (7.08)

### Q & A group session attendance

During the educational phase, one national Q&A session was offered, which was a group session open to all patients under AKT, Limited Liability Company (LLC) care including the study participants, with 8 of 40 participants (20%) attending. A second Q&A session for just LI-LI study participants was offered during the diet phase and was attended by 18 of 40 participants (45%). Among those in the diet cohort, attendance at weekly study-specific Q&A group sessions was as follows: baseline (18/25; 72%), week 1 (14/24; 58%), week 2 (11/22; 50%), and week 3 (13/21; 62%). Out of the 21 diet completers, 18 (86%) went to at least one study-specific diet group meeting.

## Main outcomes

### Self-reported competence

Overall self-reported competence on the VAS scale from 0 to 100 was *M*(*SE*) = 17.40 (2.68) before education (baseline), *M*(*SE*) = 68.08 (2.47) after education, and *M*(*SE*) = 86.62 (1.82) after the diet phase. A linear mixed model and follow-up specific comparisons showed statistically significant improvements in self-reported competence at every time point and spanning the whole study (see **Table 2**). From pre-education to post education, improvements were significant, *p* < 0.0001, with a large effect size, *d* = 2.88, 95% CI [2.17, 3.58]. From post-education to post-diet, improvements were also statistically significant, *p* < 0.0001, with a large effect size, *d* = 1.08, 95% CI [0.52, 1.61]. The total effect, spanning the whole study from pre-education to post-diet, was largest and most significant, *p* < 0.0001, *d* = 3.49, 95% CI [2.33, 4.63]. Improvements in self-reported overall competence across all three timepoints (pre-education, post-education, and post-diet) are visualized in **Figure 2**. An exploratory visualization of individual aspects of self-reported competence over the three timepoints are shown in **Figure 3** to describe specific areas of KMT which participants felt they had improved.

**Table 2:** Linear mixed model for overall self-reported competence rating at each time point.

Linear mixed model Model reference timepoint: Pre-education					
	Estimate	SE	Df	t	P-value
Intercept (pre-education)	17.40	2.39	84.16	7.29	<0.0001
Post-education	50.68	2.81	56.78	18.00	<0.0001
Post-diet	68.07	3.51	65.59	19.38	<0.0001
Specific comparisons					
Pre-education to post-education	50.7	2.81	59.4	18.01	<0.0001
Post-educatiom to post-diet	17.4	3.53	67.9	4.93	<0.0001
Pre-education to post-diet (whole study effect)	68.1	3.53	67.9	19.28	<0.0001

**Figure 2:**
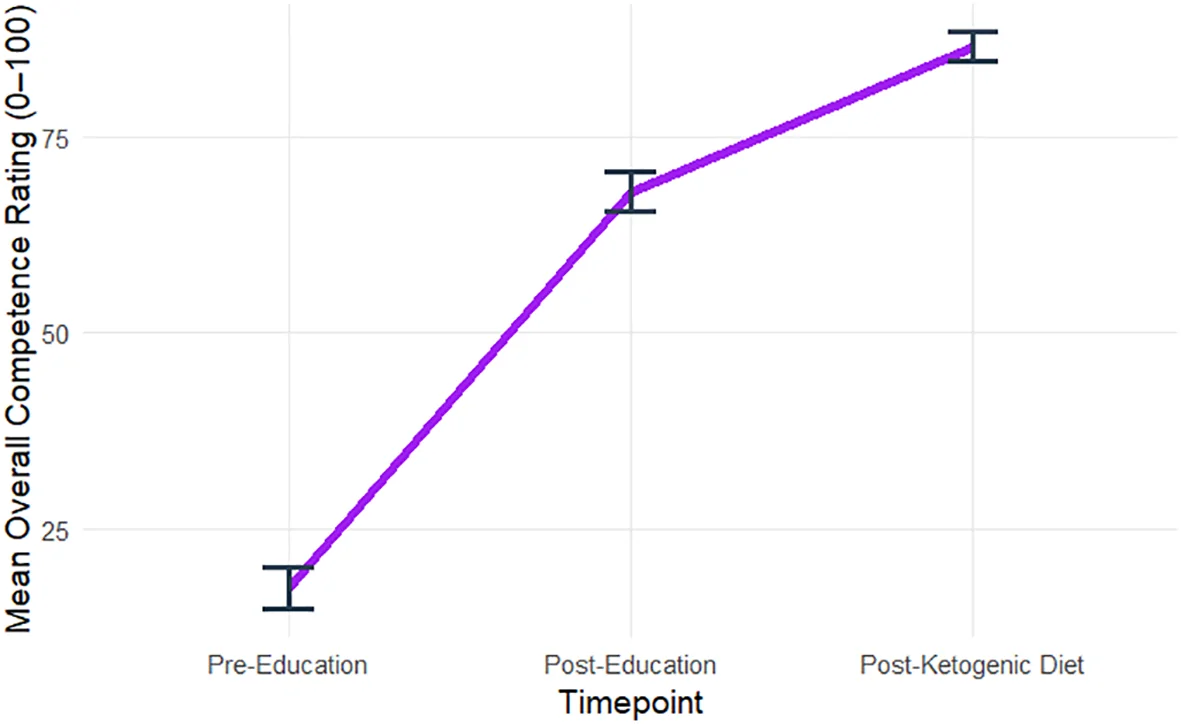
Self-reported overall competence for the implementation of a ketogenic diet (visual analog scale 0 – 100).

**Figure 3:**
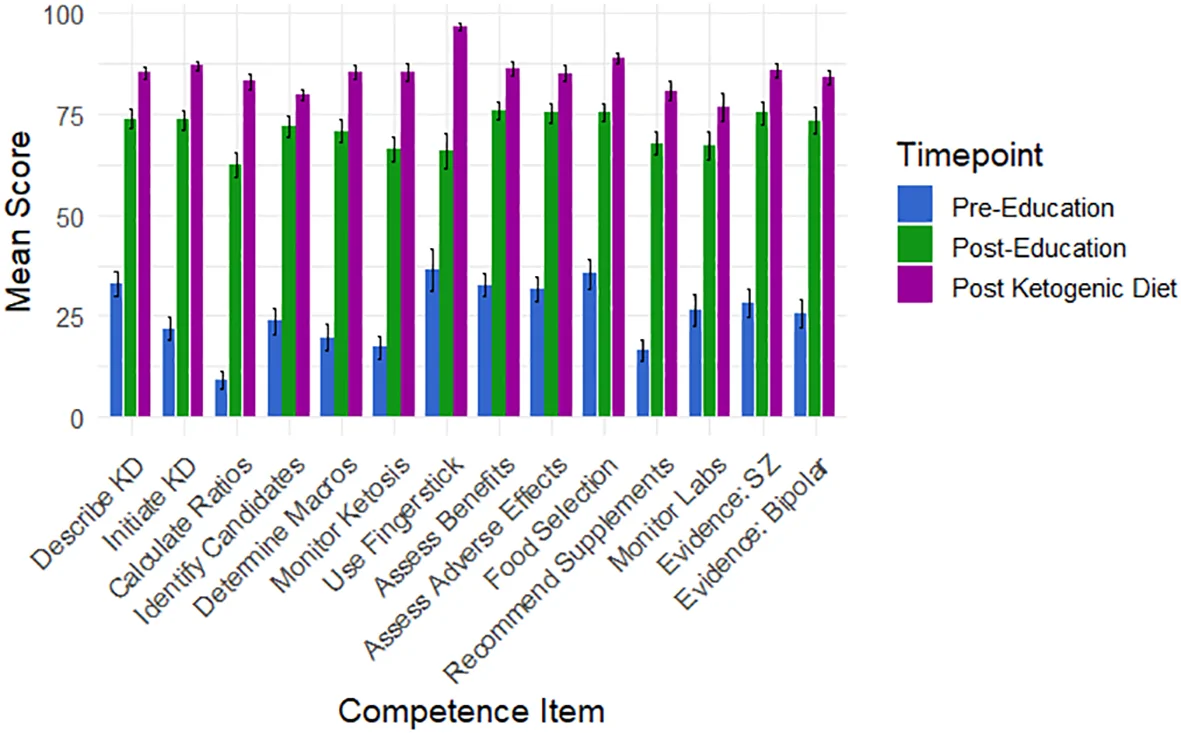
Self-reported competence for individual aspects regarding the ketogenic diet.

### Self-reported shared decision making

Perceived decision making self-efficacy to use KMT, as measured by the DSE, was *M*(*SE*) = 53.64 (2.89) at baseline. Post-education phase, average DSE was *M*(*SE*) = 83.35 (1.83). Post-lived experience on KMT, DSE level was improved to *M*(*SE*) = 91.23 (2.05). A linear mixed model and follow-up specific comparison showed statistically significant improvements in DSE at every timepoint across the whole study (See **Table 3**). Improvements from pre-education to post-education were statistically significant (*p* < 0.0001), with a large effect size, *d* = 1.74, 95% CI [1.24, 2.23]. Improvements from post-education to post-diet were statistically significant (*p* = 0.009), with a moderate-large effect size, *d* = 0.73, 95% CI [0.24, 1.20]. The total improvement in DSE scores from baseline to post-diet, was statistically significant (*p* < 0.0001), with a large effect, *d* = 2.53, 95% CI [1.63, 3.41] (See **Figure 4**).

**Table 3:** Linear mixed model for shared decision making self-efficacy score, as measured by the DSE, over all three timepoints.

Linear mixed model Model reference timepoint: Pre-education					
Predictor (time point)	Estimate	SE	Df	t-value	P-value
Intercept (pre-education)	53.64	2.28	80.94	23.54	<0.0001
Post-education	29.72	2.61	56.42	11.38	<0.0001
Post-diet	38.59	3.27	64.53	11.80	<0.0001
Specific comparisons					
	Estimate	SE	Df	T	P-value
Pre-education to post-education	29.72	2.61	59.3	11.38	<0.0001
Post-education to post-diet	8.87	3.29	67.2	2.70	0.009
Pre-education to post-diet (whole study effect)	38.59	3.29	67.2	11.74	<0.0001

**Figure 4:**
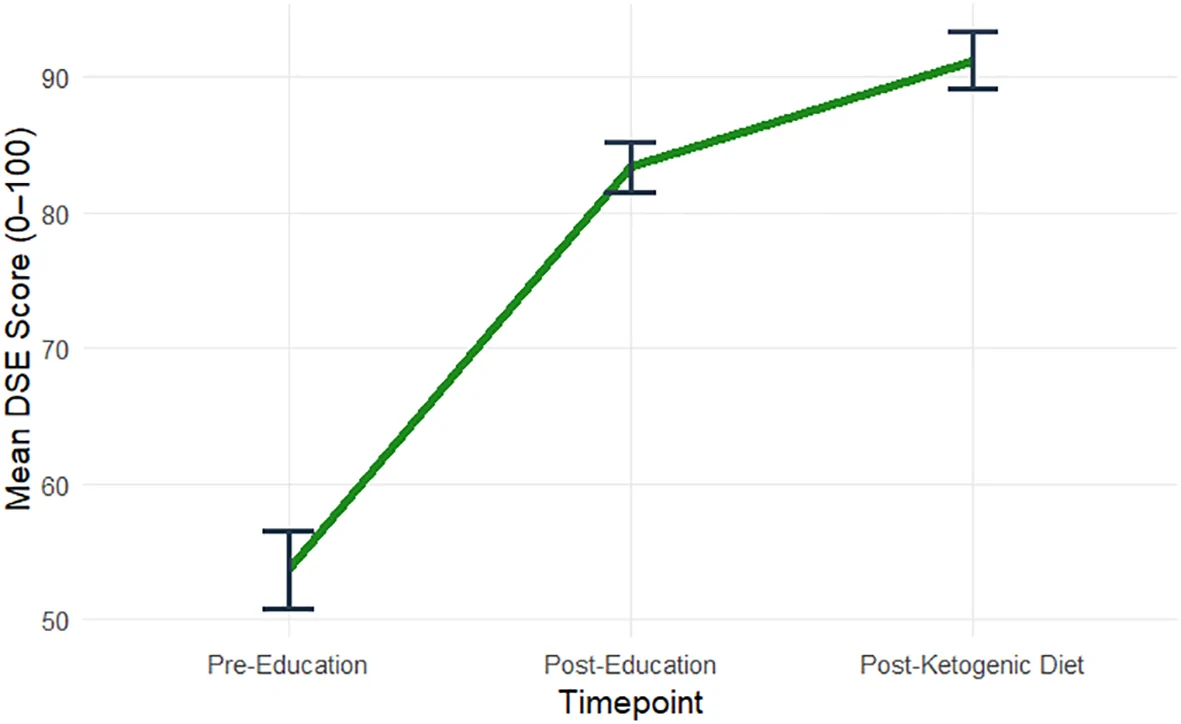
Self-reported shared decision making scores using the decision self-efficacy (dse) scale (0-100).

### Exploratory outcomes

#### Qualitative data

At study completion, 29 participants provided qualitative feedback on the program. Twenty-eight out of 29 (97%) answered “Yes” to the question “*Do you think the Live It Launch It program improved your ability to implement a ketogenic diet for your patients?”* **Supplementary Table 4** lists thematic codes identified in the open-ended qualitative responses and broader themes formed based on these codes through thematic analysis (75). These questions included helpful aspects and improvements they would make to the educational program and what discussion points they would want to emphasize with their patients, as well as expected challenges for patients implementing KMT.

Also, at the start of the study, participants completed a checklist of the clinical populations for which they may have interest in using a ketogenic diet. **Figure 5** shows the total number of HCPs (out of 40) who indicated potential interest in using KMT for each diagnosis.

**Figure 5:**
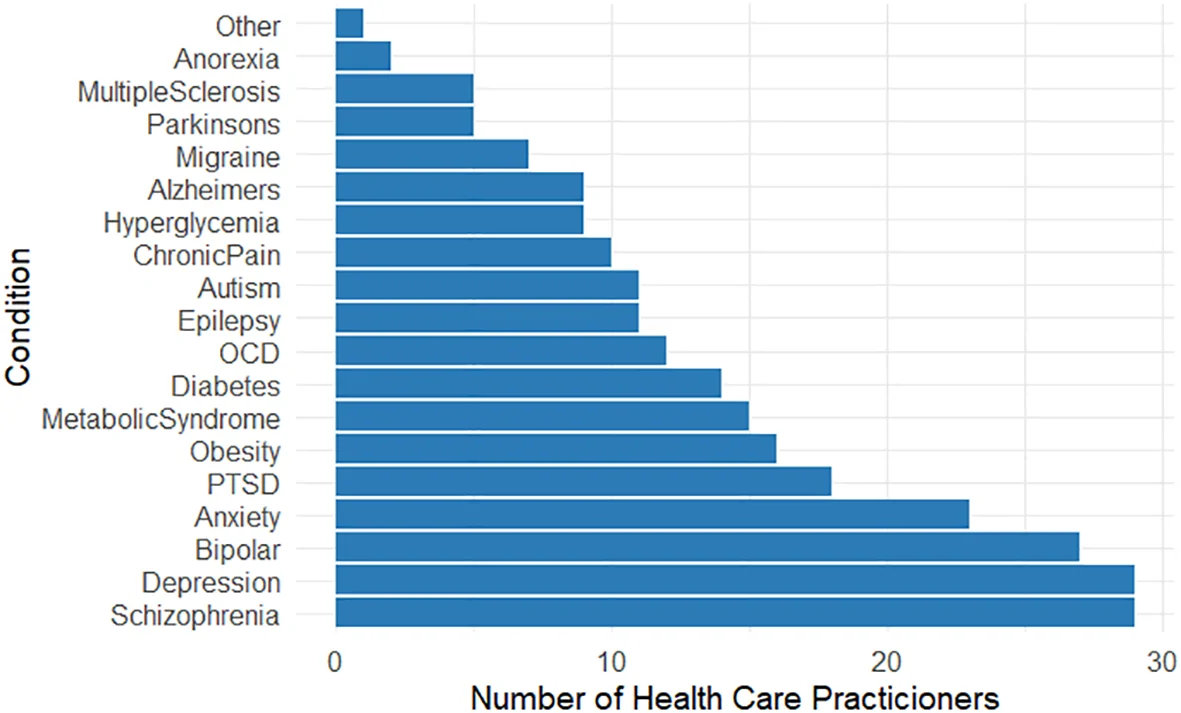
Clinical diagnoses for which health care professionals may have future interest in considering ketogenic diets for treatment (N = 40)*. *Evidence base for treatments is variable for each condition. This question provided understanding of potential future use and not a measure of efficacy, effectiveness or strength of evidence base.

## Discussion

To our knowledge, this is the first study to evaluate a KMT training program designed to improve HCP self-reported competence in introducing, offering, and discussing KMT in psychiatric care for individuals with serious mental illness. LI-LI is distinctive as it is also the first KMT training program to integrate training to enhance shared decision making in HCPs. Given the complexity of implementing dietary interventions in psychiatric care and the growing concerns that the current workforce is inadequately prepared to deliver such interventions (77), there is a need to train a variety of multidisciplinary HCPs in KMT for mental health. These findings demonstrate that LI-LI was associated with improved self-reported competence in HCPs to discuss and offer KMT and improved self-reported shared decision making capability to collaborate with patients in considering KMT as part of their treatment.

The demand for training was high, with enrollment reaching capacity within two weeks and participation spanning multiple disciplines, including physicians, pharmacists, therapists, and other HCPs. This multidisciplinary engagement is particularly encouraging given the collaborative nature of psychiatric care and ongoing workforce shortages (78–80). It is important to point out that certain HCPs can prescribe and monitor KMT themselves (e.g. physicians and RDNs), while others (e.g., psychologists, therapists, researchers) would be able to educate patients about KMT and then refer them to an appropriate specialist (e.g., RDN) for implementation and monitoring. We believe that educating people in diverse HCP roles is important, as this will increase patient reach, awareness, and knowledge of KMT, even if an additional provider is needed to monitor and implement the diet. The goal of these efforts is to ultimately increase patient access and choice, even if it takes a more collaborative care approach.

Considering the high levels of competing demands inherent to a sample of HCPs, participant engagement was strong. A slight majority completed the lived experience diet phase (21/40; 53%) and within the diet phase, most attended at least one group Q&A session (18/21; 86%).

Large and significant improvements in self-reported competence emerged after participation in LI-LI (**Table 2**). Meaningful changes on VAS are often in the range of 13–15 points (81–84), so the observed increase of 69.2 points overall represents a substantial effect. Mean self-reported overall competence scores were ~87 after the lived diet experience, signifying high self-perceived confidence in the learned skills. The data suggests that both KMT education and self-implementation were important for increasing HCP self-reported competence to use KMT with patients in the future.

Parallel improvements were observed in self-reported shared decision making skills for discussing and/or implementing a ketogenic diet (**Table 3**). It is important for HCPs to be able to engage patients and families in informed, collaborative treatment discussions, and thus we wanted to provide pilot data in this area as it related to their confidence in implementing discussions of KMT for treating mental health disorders. Preparing HCPs to have the skills and confidence to work with their patients is essential. Previous work has demonstrated that individuals initiating KMT require time to develop an understanding of carbohydrate content in foods, how these foods influence blood glucose and ketone levels, and how to successfully achieve and maintain nutritional ketosis (85). This initial learning period highlights that the complexities of KMT are not immediately intuitive, and a training model like LI-LI may help to provide skills necessary for HCPs to support patients in a collaborative fashion as they navigate KMT. Our inclusion of a shared decision making scale (the DSE) was unique in that most KMT interventions are aimed at families and patients, but less so on the clinician’s ability to engage in this important treatment aspect (86).

Regarding shared decision making, participants identified several important considerations for future patient education and clinical implementation. Experiencing the diet firsthand increased awareness of common adherence barriers and reinforced the importance of proactively discussing strategies to support long-term adherence with patients. Many participants reported feeling confident in explaining KMT. However, they also recognized the value of involving an RDN, and the need to do so when the intervention extends beyond their professional scope of practice. The thematic findings also reinforce that successful implementation requires presenting a balanced perspective that communicates both the potential benefits and practical challenges of KMT, enabling patients to develop realistic expectations and facilitating informed, shared decision making. These findings align with previous work on shared decision making (87, 88), conceptualizing shared decision making as an ongoing therapeutic partnership that is strengthened by incorporating the lived experiences and perspectives of both service users and clinicians and by openly addressing barriers and facilitators to treatment implementation. Our findings extend these concepts beyond mental health and to clinician education by suggesting that incorporating lived experience into KMT training may improve clinicians’ communication skills, confidence, and ability to engage patients in collaborative treatment decisions. Future work should focus on developing evidence-based approaches for discussing and implementing KMT with individuals living with severe mental illness, as their clinical, cognitive, and psychosocial needs may require tailored communication and support strategies.

The qualitative analyses also provided insight into pros and cons of the pilot program. Overall, participants reported high satisfaction with both the educational curriculum and the lived-experience component, with many indicating intentions to either implement KMT in practice or refer appropriate patients, suggesting potential downstream clinical impact. Thematic analyses demonstrated that both the didactic coursework and lived-experience diet contributed meaningfully to learning, while weekly group support sessions with dietitians and peers facilitated adherence and fostered a sense of comradery similar to that reported among patients using KMT (89). Participants’ firsthand experience also highlighted practical challenges that are now better understood by both the research team and participating health care professionals. Recommendations for improving the LI-LI program included providing more information about potential side effects, particularly long-term lipid effects, streamlining and updating the educational modules, and more clearly defining the roles of different health care professionals in implementing KMT versus educating and referring patients to physicians or registered dietitians.

### Limitations and future directions

The self-reported competence and shared decision making measures do not equate to objective or applied skills or to behavioral changes associated with implementation. Future testing of an updated training model should include objective implementation outcomes, such as measuring frequency of discussions of KMT, patient education, referrals to registered dietitians and metabolic psychiatry services, and prescription/implementation of KMT when clinically appropriate.

The current study design was a small single arm trial in which several study team members were also study participants. Further, it is important to mention that a smaller sample (N = 26) elected to try the diet phase out of the N = 40 who completed the education. Therefore, not everyone participated in experiential learning. Data from study team members were included in all analyses. Due to the small sample size, sensitivity analyses excluding study team participants were not performed. Author SLM analyzed the data, and the research coordinator recruited participants and collected the data. Therefore, due to several members of the study team participating as subjects, this study is not free from bias, and results should be interpreted with caution, including the magnitude of the effect sizes.

Given also that the diet phase of the study was optional, the diet sample was self-selected and is prone to selection bias, likely towards those with special interest in, or positive inclination towards, KMT. Therefore, findings in the diet phase should be interpreted as exploratory. Furthermore, these study results do not suggest the education course used is exclusive, as other courses on the topic are available. Data and feedback from this study will be used to inform an updated approach to providing KMT education specifically designed for health care professionals providing care to psychiatric patients.

Importantly, the diagnostic groups for which participants indicated consideration in using KMT merely reflect participants’ reported interest. Each illness listed in **Figure 5** has undergone or is undergoing some level of research with KMT; however, evidence for the use of KMT in most of these illnesses is in an early stage far from determining the appropriateness of widespread use.

We recommend that future KMT training models for HCPs, like LI-LI, include an expert in KMT delivery on the team, such as a registered dietitian, and additional access to medical providers for monitoring and side effect management. Also, trained educators and implementation scientists should be included in future projects using and studying an updated version of this educational approach. We also believe the next steps should incorporate objective outcome measures of behavior change and larger participant sample sizes to test the reach and impact of this training approach for HCPs.

## Conclusion

The present study was a single-arm, open-label pilot trial training HCPs about KMT as an adjunct treatment for mental illness. As this, to our knowledge, was the first study of its kind, training outcomes were limited to self-reported measures of HCPs' perceived competence to introduce, discuss, and implement KMT with patients and perceived self-efficacy to collaborate with patients through shared decision making about incorporating KMT into a treatment plan. Participants demonstrated statistically significant improvements in both self-reported competence and perceived shared decision making self-efficacy. However, this was a small pilot feasibility study in which several members of the study team also participated, and the findings should therefore be interpreted with caution. Nevertheless, these preliminary quantitative and qualitative findings support further refinement of the training program, evaluation in a larger sample, and inclusion of more objective outcome measures to determine whether an updated version of this model can serve as an effective approach for building HCP capacity to use KMT. Our work reinforces that future implementation of evidence-based treatments requires more than passive knowledge acquisition; it depends on building competence, confidence, and practical skills.

## Data Availability

The raw data supporting the conclusions of this article will be made available by the authors, without undue reservation.
