# Supplementary material for: Pilot evaluation of an educational and lived experience training program on ketogenic metabolic therapy in mental health care for health care professionals
Source: Front Psychiatry. 2026 Sep 22;17:1913325. doi: 10.3389/fpsyt.2026.1913325 (PMC13638670; doi:10.3389/fpsyt.2026.1913325)
Supplement: Supplementary file 1 [file Table1.docx]

**Supplemental Tables**

Supplemental Table 1. Details of the Educational Program

| Module | Section | Title | Duration | Description |
| --- | --- | --- | --- | --- |
| Module 1: Foundations of Ketogenic Metabolic Therapy | | | | |
|  | 1 | History of KMT and Psychiatric Applications | 10 min | Review the history and development of ketogenic metabolic therapy (KMT), including emerging evidence for its application in psychiatric and mental health conditions. |
|  | 2 | Macronutrients and Metabolism | 10 min | Review ultra-processed foods, carbohydrate intake, and NIH recommendations for protein, fat, and carbohydrate consumption. Describe glucose responses and glucose curves observed with various dietary patterns. |
|  | 3 | Brain Nutrition | 2 min | Describe nutritional deficiencies that may contribute to symptoms of depression and other mental health concerns. |
|  | 4 | Ketosis Defined | 10 min | Review therapeutic ketosis and the biochemistry of ketone production and utilization, including its effects on the body. Describe ketone measurement methods, fasting considerations, and the role of exogenous ketones. |
|  | 5 | Practice Guidelines | 18 min | Review clinical considerations for implementing KMT in mental health, including medications, comorbidities, diet prescriptions, adverse effects, screening, assessment, laboratory testing, diet preparation, personalization, and ongoing support. Review the five primary variations of ketogenic metabolic therapies. |
| Module 1 Total |  |  | **50 min** |  |
| Module 2: Variations and Personalization of Ketogenic Metabolic Therapy | | | | |
|  | 1 | Variations of KMT | **30 min** | Provide an in-depth review of the five primary variations of ketogenic metabolic therapies, including the Classic Ketogenic Diet, Modified Ketogenic Diet, Modified Atkins Diet, MCT Oil Diet, and Low Glycemic Index Treatment. Review key features, applications, and ketogenic ratios used within these approaches. |
|  | 2 | Choosing a KMT | **7 min** | Review the rationale and clinical considerations for selecting specific ketogenic metabolic therapies  based on individual patient needs, goals, medical conditions, and lifestyle factors. |
|  | 3 | Personalizing KMT | **2 min** | Describe the importance of individualizing ketogenic therapy, including diet selection, supplementation, hydration, fat intake, metabolic markers, and expected timelines for adaptation and therapeutic response. |
|  | 4 | Case Study: Schizoaffective Disorder | **14 min** | Review a case study of an individual with schizoaffective disorder who experienced significant weight gain associated with psychiatric medications and severe functional limitations, including near-agoraphobia. Follow the patient’s nine-month ketogenic therapy journey and examine clinical, metabolic, and quality-of-life outcomes. |
| Module 2 Total |  |  | **53 min** |  |
| Module 3: Well-Formulated Ketogenic Diet | | | | |
|  | 1 | KMT Review | **3 min** | Provide a brief review of ketogenic metabolic therapy principles, including ketogenic ratios, therapeutic goals, and the impact of macronutrients on glucose metabolism and ketosis. |
|  | 2 | Dietary Fats in Ketogenic Therapy | **12 min** | Review the role of dietary fats in ketogenic metabolic therapy, including monounsaturated, polyunsaturated, and saturated fats. Describe omega-3 and omega-6 fatty acids, common dietary oil varieties and their benefits, the therapeutic applications of MCT oil, and considerations regarding the use of butter and other fat sources within ketogenic diets. |
|  | 3 | Carbohydrates, Fiber, and the Gut Microbiome | **17 min** | Describe the role of carbohydrates, fiber, and the gut microbiome in health and ketogenic therapy. Review prebiotics, probiotics, polyphenols, and strategies that support a healthy gut microbiome. Describe intestinal permeability ('leaky gut') and factors that may negatively affect gut health and microbial diversity. |
|  | 4 | Protein | **6 min** | Review protein requirements, including the Recommended Dietary Allowance (RDA), amino acids, and the impact of protein intake on ketogenic metabolic therapy and metabolic health. |
|  | 5 | Supplementation | **15 min** | Review the importance of obtaining micronutrients from whole foods whenever possible,  highlighting nutrient-dense food sources commonly used in ketogenic therapy. Describe common  supplements utilized in ketogenic metabolic therapy, indications for use, and practical  supplementation considerations. |
| Module 3 Total |  |  | **53 min** |  |
| Module 4: Ketogenic Management | | | | |
|  | 1 | Medications | **11 min** | Review healthcare provider responsibilities related to ketogenic metabolic therapy, including medication management, deprescribing considerations, and the importance of monitoring and adjusting treatments for comorbid medical and psychiatric conditions as appropriate. |
|  | 2 | Monitoring | **14 min** | Review home-based and laboratory monitoring strategies used during ketogenic metabolic therapy. Describe ketone monitoring methods, including blood, urine, and breath ketone testing, as well as glucose monitoring approaches such as finger-stick glucose testing and continuous glucose monitoring (CGM). |
|  | 3 | Laboratory Monitoring | **3 min** | Review recommended laboratory evaluations, interpretation considerations, and suggested schedules for laboratory monitoring during ketogenic metabolic therapy. |
|  | 4 | Adverse Effects | **34 min** | Describe the eight most common potential adverse effects associated with ketogenic metabolic therapy and evidence-based strategies to prevent, identify, and mitigate these effects while supporting long-term adherence and safety. |
| Module 4 Total |  |  | **62 min** |  |

Supplemental Table 2. Individual Items for Self-Reported Competence (14 items)

| **Self-Reported Competence Scale**  **(100 mm Visual Analog Scale)** | |
| --- | --- |
| How competent are you in describing how a ketogenic diet works? | Not competent at all Somewhat competent Extremely Competent |
| How competent are you in explaining/demonstrating how to initiate a medical ketogenic diet? | Not competent at all Somewhat competent Extremely Competent |
| How competent are you in calculating keto diet ratios for a patient following a ketogenic diet/therapy? | Not competent at all Somewhat competent Extremely Competent |
| How competent are you in determining who would benefit from/who is appropriate for a medical ketogenic diet? | Not competent at all Somewhat competent Extremely Competent |
| How competent are you in determining the macronutrient composition of a ketogenic diet? | Not competent at all Somewhat competent Extremely Competent |
| How competent are you in monitoring ketosis and making dietary adjustments as needed? | Not competent at all Somewhat competent Extremely Competent |
| How competent are you in using the fingerstick point of care device to monitor ketosis? | Not competent at all Somewhat competent Extremely Competent |
| How competent are you assessing the benefits of a ketogenic diet? | Not competent at all Somewhat competent Extremely Competent |
| How competent are you in assessing the adverse effects of a ketogenic diet? | Not competent at all Somewhat competent Extremely Competent |
| How competent are you in recommending selecting/shopping for food that will make up a ketogenic diet? | Not competent at all Somewhat competent Extremely Competent |
| How competent are you in recommending vitamin/other supplementation for a ketogenic diet/therapy? | Not competent at all Somewhat competent Extremely Competent |
| How competent are you at monitoring lab work for a patient on a ketogenic diet/therapy? | Not competent at all Somewhat competent Extremely Competent |
| How competent are you in relaying the literature and evidence for a ketogenic diet for schizophrenia? | Not competent at all Somewhat competent Extremely Competent |
| How competent are you in relaying the literature and evidence for a ketogenic diet for bipolar disorder? | Not competent at all Somewhat competent Extremely Competent |

Supplemental Table 3. Decision Self-Efficacy (DSE) Scale

| **Decision Self-Efficacy (DSE) Scale** | |
| --- | --- |
| Definition: The scale measures self-confidence or belief in one’s abilities in decision making including shared decision making. | |
| **My confidence in supporting my patients making an informed choice**  **regarding a ketogenic diet**  Below are listed some things involved in supporting patients in making an informed choice regarding Keto diet.  Please show how confident you feel in doing these things by circling the number from 0 (not at all confident) to 4 (very confident) for each item listed below. | |
| **I feel confident that I can:** |  |
| 1. Get my patients the facts about the diet choices available to them | 0 1 2 3 4  Not at all confident Very confident |
| 2. Get my patients the facts about the benefits of each choice | 0 1 2 3 4  Not at all confident Very confident |
| 3. Get my patients the facts about the risks and side effects of each choice | 0 1 2 3 4  Not at all confident Very confident |
| 4. Help my patients understand the information enough to be able to make a choice | 0 1 2 3 4  Not at all confident Very confident |
| 5. Encourage my patients to ask me questions without making them feel dumb | 0 1 2 3 4  Not at all confident Very confident |
| 6. Help my patients feel comfortable to express their concerns about each choice | 0 1 2 3 4  Not at all confident Very confident |
| 7. Support my patients to ask me and others for advice | 0 1 2 3 4  Not at all confident Very confident |
| 8. Help my patients to figure out the choice that best suits them | 0 1 2 3 4  Not at all confident Very confident |
| 9. Help my patient to handle unwanted pressure from others, including from myself, in making their choice | 0 1 2 3 4  Not at all confident Very confident |
| 10. Support my patients in letting others who treat them to know what's best for them | 0 1 2 3 4  Not at all confident Very confident |
| 11. Make my patient feel comfortable with delaying their decision if they need more time. | 0 1 2 3 4  Not at all confident Very confident |

Supplemental Table 4. Thematic Analysis of Qualitative Questions about Educational Outcomes

| **Research Question** | **Initial Codes** | **Candidate Themes** | **Final Themes** | **Illustrative Quotes** |
| --- | --- | --- | --- | --- |
| What aspect(s) of the program helped you learn the best? Did you learn anything that surprised you? | 1. Meetings with dietitian 2. Diet implementation 3. Educational modules (general) 4. How to calculate macronutrient ratios 5. The variety of ketogenic diets 6. Tracking via bloodwork/fingerstick 7. Group meetings | 1. Educational coursework helped participants learn   (codes: 3, 4, 5)   2. Diet implementation helped participants learn   (codes: 2, 4, 6)   3. Supportive and informational meetings helped participants learn   (codes: 1, 7) | (Candidate themes remained)   1. Educational coursework helped participants learn (codes: 3, 4, 5) 2. Diet implementation helped participants learn (codes: 2, 4, 6) 3. Supportive and informational meetings helped participants learn (codes: 1, 7) | “I feel more comfortable and confident in assisting a patient to implement a ketogenic diet. The initial training materials and the times with the dietitian greatly enhanced my knowledge and confidence. The most significant attribute was experiencing the diet myself.”  (Themes: 1, 2, 3) |
| What, if anything, would you change about the education portion? | 1. More information on safety & supplementationon KD 2. More information on physiology of KD 3. More information on KD application 4. Streamline and shorten educational modules 5. Reorganize educational modules 6. Provide CME credits 7. Criticism of the strength or date of presented data 8. Criticism of including too much epilepsy data 9. More guidance on professional scope related to implementing the diet 10. Off topic responses (discussing diet phase rather than education) 11. Satisfied with educational portion, no changes | 1. Request for more information on KD 2. Satisfied with educational portion, no changes 3. Streamline & Organize Educational Modules 4. Application for more time to digest the material 5. Professional Scope Concerns with Implementing the diet 6. Criticism of presented evidence | 1. Request for more information on KD   (codes: 1, 2, 3)   2. Request to streamline & organize modules   (codes: 4, 5)   3. Criticism of presented data   (codes: 7, 8)   4. Professional scope concerns with implementing the diet   (code: 9) | “I would ask an MD to create slides to further clarify risk of heart disease with a long-term ketogenic diet.” (Theme 1)  “It could be helpful to divide it into two parts...Part 1 could be a collection of evidence regarding the ketogenic diet and mental health, and Part 2 could be the actual how-to training. That, way it’s organized, where people can understand the “why,” ...but if they’re more interested in the “how,” ...they can go to that section.” (Theme 2) |
| How will you implement the ketogenic diet for your patients? What aspects of the diet will you emphasize to them?  What do you foresee being the easiest and most difficult parts of the diet to explain or implement with patients? | 1. Cost is a barrier for psychiatric patients 2. Planning is a barrier for psychiatric patients 3. Limited professional scope; need for RDN 4. Specific go-to strategies 5. Confident to explain KD 6. Emphasize importance of adherence and diet as medical treatment 7. Explain potential benefits for disease state(s) 8. Emphasize self-monitoring of ketones, glucose, food 9. Use of tools 10. Preparing ahead of time/slow ramp up period | 1. Barriers to adherence 2. Emphasize   appropriate  macronutrient  ratios   3. Emphasize self-monitoring and adherence 4. Offer specific go-to strategies 5. Use of tools 6. Limited professional scope to implement KD/Need for RDN 7. Feel confident to explain KD to patients | 1. Barriers to adherence   (codes: 1, 2)   2. Emphasize importance/ potential benefits of adhering   (codes 6, 7)   3. Emphasize  supportive strategies for adherence   (codes: 4, 8, 9, 10)   4. Limited Professional Scope to Implement KD/ Need for RDN   (code: 3)   5. Feel confident to explain KD to patients   (code: 5) | “Emphasizing self-monitoring (meter or other) and need to adhere to the diet...challenges with access to keto friendly foods and cost for patients. I found the diet to be expensive to start up.” (Themes: 1, 2, 3)    “A slow transition was important for me...and carry a source of fat with you like MCT oil if you want to eat out. Working with a dietitian is important. You may feel better which will make it easier to keep going. The difficult part is just planning ahead and making sure you’re drinking enough water.”  (Themes: 1, 2, 3, 4) |
